# P71 A qualitative evidence synthesis to explore the patient experience of antimicrobial resistance

**DOI:** 10.1093/jacamr/dlag102.077

**Published:** 2026-06-26

**Authors:** Emma Kearney, Karen Fitzmaurice, Aoife Fleming

**Affiliations:** School of Pharmacy, University College Cork, Cork, Ireland; Microbiology Department, Cork University Hospital, Cork, Ireland; School of Pharmacy, University College Cork, Cork, Ireland; Pharmacy Department, Mercy University Hospital, Cork, Ireland

## Abstract

**Background:**

The increase of antimicrobial resistance (AMR) contributes to significant morbidity and mortality internationally. At an individual patient level, AMR can have a devastating impact on patient health in the short and long term. Understanding the consequences on patients’ quality of life, physical and psychological health is important to capture the full effect of AMR. The aim of this study was to conduct a qualitative evidence synthesis to understand patients’ experiences of AMR and its impact on their quality of life.

**Methods:**

A comprehensive search of three electronic databases (PubMed, CINAHL, Web of Science) was conducted (July 2025) to identify qualitative studies that reported the experiences of adults with AMR infection or colonization. Peer-reviewed studies using an interview or mixed method design with qualitative components were included. Studies including patients from any healthcare setting with experiences of multi or single drug resistance, from any source infection, were considered. Quality appraisal of studies was assessed using the Critical Appraisal Skills Programme (CASP) assessment tool for qualitative research. The Thomas and Harden approach to inductive thematic synthesis was adopted.

**Results:**

Twenty-eight studies (2001–25) were included from ten countries, addressing a range of AMR infections such as Methicillin Resistant *Staphylococcus aureus*, *Clostridioides difficile*, Extended-spectrum β-lactamases and Carbapenemase Producing Enterobacterales. The four main interconnected themes conveyed the impact of AMR on patients; (i) Burden of infection, AMR and treatment, (ii) Identity and stigma, (iii) Isolation experience and (iv) Information, communication and healthcare practices. The physical and social isolation experienced and the health burden of infection experienced were compounded by gaps in communication and inconsistencies in healthcare practices. This was found to lead to reduced understanding and patients feeling afraid and losing trust in the healthcare system.

**Conclusions:**

This qualitative synthesis highlights the distressing physical and psychological impact of AMR experienced by patients including social isolation, uncertainty, alienation, and reduced quality of life. The findings highlight the need for support for patients and their families, and enhanced education and training for healthcare professionals, to enable patient understanding, recovery and rehabilitation.

